# Analysis of HSV1/2 Infection Reveals an Association between HSV-2 Reactivation and Pregnancy

**DOI:** 10.3390/v16091370

**Published:** 2024-08-28

**Authors:** Sara Dovrat, Adar Shabat, Anat Yahav-Dovrat, Zvia Soufiev, Ella Mendelson, Ela Kashi-Zagdoun, Galia Rahav

**Affiliations:** 1 National Center for Herpes, Central Virology Laboratory, Ministry of Health, Sheba Medical Center, Ramat Gan 52621, Israel; saradovrat@gmail.com (S.D.);; 2 Department of Radiology, Rambam Health Care Campus, Haifa 31096, Israel; 3 Infectious Diseases Unit, Sheba Medical Center, Ramat Gan 52621, Israel; 4 Sackler Faculty of Medicine, Tel-Aviv University, Tel Aviv 69978, Israel

**Keywords:** HSV1, HSV2, progesterone, pregnancy, gender, reactivation

## Abstract

The herpes simplex viruses consist of the strains, HSV-1 and HSV-2, which are prevalent worldwide and lack a definitive cure. We aimed to explore the specific characteristics of HSV 1 and 2 infections, such as differences between gender assigned at birth, age at infection, site of infection, comorbidities, and effect of pregnancy, through a data analysis. Between 2011 and 2018, the Israeli Central Virology Laboratory diagnosed 9189 samples using multiplexed real-time PCR. In addition, we extracted all of the medical data for 287 females hospitalized at the Sheba Medical Center with HSV-1 (161) or HSV-2 (126) genital infections. HSV-2 was almost absent in the orofacial samples from both genders, while in other lesion sites, HSV-2 was significantly more abundant in females than in males (*p* < 0.05,). HSV-2 was initially detected at puberty. In the hospitalized females’ malignancies, both HSV-1 and HSV-2 were found with a non-significant difference. Simultaneously, pregnancies were more common in females who were HSV-2-positive compared with those who were HSV-1-positive (27.8% vs. 12.4%, respectively, *p* < 0.01). Primary infections occur more with HSV-1 than with HSV-2 (15.6% vs. 3.2%, respectively). Our findings demonstrate that genital HSV-2 infection episodes are more frequent during pregnancy, suggesting that pregnancy may serve as a risk factor for HSV-2 reactivation or infection.

## 1. Introduction

The herpes simplex virus-1 (HSV-1) and HSV-2 belong to the alpha herpes viruses subfamily which is characterized by a short replication cycle, lysis of the host cell, and the establishment of latency in the sensory ganglia, where they can be reactivated [1]. HSV-2 was previously considered to be the predominant subtype of genital infections.

However, HSV-1 has been now recognized as an increasing cause of genital infections and accounts for more than 50% of new infections, mainly among young women and men who have sex with men [2,3]. Women are more susceptible to HSV-2 infections than men [4].

An HSV-2 infection enhances coinfection with other viruses. HSV-2 increases the risk of acquiring human immunodeficiency virus (HIV) and a recent study even reported an association between the selection of HIV variant antiretroviral resistance mutations and HSV-2 infection [5].

Another study reported that an HSV-2 infection enhances placental sensitivity to Zika virus (ZIKV) by enhancing the expression of TAM receptors (Tyro3, Axl, and Mer), which facilitate ZIKV cell entry [6].

In addition to above mentioned risks, genital HSV infections are of particular concern in pregnant women because of the risk of transmission to the infant during delivery [4,7]. The Israeli Central Virology Laboratory at Sheba Medical Center serves as the National Center of Herpes Viruses and routinely examines samples for HSV-1 and HSV-2 that are obtained from various sites. During 2011–2018, 11,091 samples were obtained from 9189 patients.

In this study, we have described the distribution of HSV-1 and HSV-2 infections classified by age, gender at birth, and site of infection. In addition, we have characterized the features of HSV-1 vs. HSV-2 genital infections among hospitalized women.

## 2. Materials and Methods

### 2.1. Study Design and Participants

During 2011–2018, the National Center for Herpes Viruses processed 9189 patient- specific samples suspected of HSV infection, obtained from 4727 hospitalized patients (51.4%) and 4462 outpatients (48.5%). Samples were obtained from orofacial sites which included the oral cavity, oropharynx, nasopharynx, eyes, and bronchoalveolar lavage (BAL), and genital sites which included the vagina, cervix, buttock, rectal area, and penis. Other sites included mainly skin samples that were not associated with orofacial or genital lesions. The study also included an analysis and comparison of medical records between a cohort of 161 women hospitalized at SMC with HSV-1 genital infections and a cohort of 126 women hospitalized at SMC with HSV-2 genital infections.

### 2.2. Laboratory Assay

Swabs (Viticulture, Sigma-Aldrich, Rehovot, Israel) collected from the various sites described were examined for HSV-1 and HSV-2 presence using multiplex real-time PCR (qPCR) as described below.

### 2.3. DNA Extraction

Viral genomic DNA was extracted using an automatic DNA extractor (MagNa Pure Roche Molecular Biochemical, Indianapolis, IN, USA), following the manufacturer’s instructions.

### 2.4. TaqMan Real-Time (RT) PCR

The ABI PRISM 7500 sequence detection system (Applied Biosystems, Foster City, CA, USA) was used for the amplification and detection of genomic DNA sequences. HSV-1 and HSV-2 primers and probes were designed as previously described [8]. The tests were performed with a 25 μL reaction volume, using the TaqMan master mix (Eurogentec RT-QP2X03-50), TaqMan primers (300 nM per reaction), labeled probe (200 nM), and 10 μL of DNA extract. RT-PCR was performed under the following conditions: 2 min at 50 °C, 10 min at 95 °C, 50 cycles of 15″ at 95 °C, and 1 min at 60 °C.

### 2.5. Statistical Analysis

Means ± SD were calculated for continuous variables; absolute and relative frequencies were measured for discrete variables. Differences in means and proportions were assessed using the Student t-test and Chi-squared test, for categorical and continuous data, respectively. Univariate logistic regression analyses were performed to identify significant covariates associated with the type of HSV. Covariates that were found to be associated with HSV-1 or HSV-2 infections, were applied in a stepwise multivariate logistic regression analysis. Odds ratios (ORs) and 95% confidence intervals (CIs) were calculated in the final models. All tests of significance were two-tailed. A value of *p* < 0.05 was considered statistically significant. All statistical analyses were performed using Statistical Analysis System (SAS), Version 9.4 (SAS Institute, Cary, NC, USA).

## 3. Results

### 3.1. Sample Analysis According to Gender and Lesion Site

Between 2011 and 2018, 11,091 samples were obtained for HSV testing from 9189 patients; 4727 were obtained from hospitalized patients and 4462 were obtained from outpatients. A total of 28.4% (*n* = 2615) had a positive result. Out of those who tested positive, 756 were men and 1859 were women. Of the samples, 2299 were obtained from orofacial sites, 2247 were obtained from genital sites, and 4643 were obtained from other sites (Table 1).

A total of 28.8% (*n* = 663/2299) of the orofacial samples tested positive for HSV, 96.3% (639/663) for HSV-1, and 3.6% (24/663) for HSV-2. No difference was found in the distribution of orofacial HSV-1 and HSV-2 among men or women (Table 1).

A total of 29.7% (*n* = 669/2247) of the genital samples tested positive for HSV, 53.3% (357/669) for HSV-1, and 46.6% (312/669) for HSV-2. Women had similar frequencies of genital HSV-1 and HSV-2, 16.0% (332/2079) and 14.4% (300/2079), respectively, but significantly higher detection rates of HSV-2 compared with men, 7.1% (12/168) (Table 1, *p* < 0.05). Men had a higher frequency of genital HSV-1 infections compared with HSV-2 infections, 14.8% (25/168) and 7.1% (12/168), respectively (*p* < 0.05).

A total of 27.6% (*n* = 1283/4643) of the samples obtained from other sites were HSV positive; 69.6% (894/1283) were HSV-1 and 30.3% (389/1283) were HSV-2. The frequency of HSV-1 infection was similar between men and women, while HSV-2 had significantly higher detection rates among women (10.0% and 4.4%, respectively) (Table 1, *p* < 0.0001).

### 3.2. Sample Analysis According to Gender and Age of Onset

**Figure 1:**
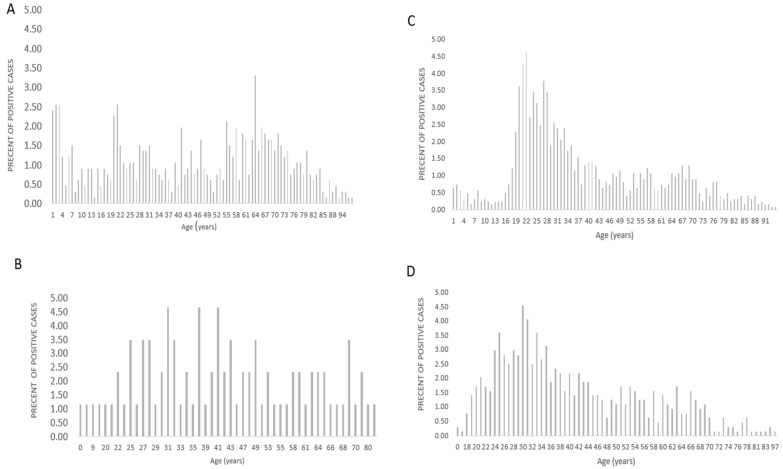
Distribution of HSV-1/2 infection by age and gender. The percentage of HSV-1/2-positive cases has been plotted by gender and age at the time of referral. (**A**) Percentage of HSV-1 cases by age in men. (**B**) Percentage of HSV-2 cases by age in men. (**C**) Percentage of HSV-1 cases by age in women. (**D**) Percentage of HSV-2 cases by age in women.

We plotted the percentages of HSV-1/2-positive cases according to gender and age at the time of referral. HSV1 and 2 infections in women showed a bell-shaped curve with peak HSV-1 and HSV-2 infections occurred between the ages of 20 and 28 and 24 and 43 years, respectively, (Figure 1C,D), while HSV1 and 2 infections in men displayed sparse acquisition with no age-defined peak (Figure 1A,B). HSV-1 was detected in early childhood, whereas HSV-2 was detected at later ages in both genders.

### 3.3. Characterization of Genital Infections among Women

Genital HSV-1 and HSV-2 infections were detected in 161 and 126 hospitalized women, respectively, for whom medical data were available and are summarized in Table 2. The mean age was 31.5 years for those with HSV-1 and 41.2 years for those with HSV-2 (*p* < 0.0001). Primary infections, defined as cases where recurrency was not documented, had significantly higher detection rates among those with HSV-1 infection (15.6%) compared to those with HSV-2 (3.2%) (*p* = 0.0005). Of the hospitalized patient cohorts, 55 women were pregnant, 12.4% (20/161) with HSV-1 infection, and 27.8% (35/126) with HSV-2 (*p* = 0.001). Miscarriage up to 6 months before infection occurred in 4 women with HSV-2 infections (4/126, 3.2%) but were not observed in the HSV-1 infection cohort (0/161). Birth control pills were taken by 26.8% of the cohort, 36.6% of those with an HSV-1 infection compared to only 14.3% of those with an HSV-2 infection (*p* < 0.0001). There was no difference in the distribution of HSV-1 or HSV-2 among those who underwent in vitro fertilization (IVF).

Multivariate logistic regression analysis found that risk factors for genital HSV-2 infection in our cohort were non-primary infection (OR 4.1, 95% CI, 3.4–12.5), pregnancy (OR 3.52, 95% CI, 1.84–6.73), and age (OR 1.23, 95%CI, 1.13–134). The odds ratio to acquiring HSV-2 infection increased by a factor of 1.23 for each 5-year increase in age (Table 3).

## 4. Discussion

We found that 28.4% of the suspected HSV infections were eventually positive. The rate was different between the two types. HSV-1 was detected in 20.5% of the samples while HSV-2 was much less abundant and was found in only 7.8% of them. Others found frequencies of 47.8% and 11.9%, respectively [9]. A large cohort study from South Korea found frequencies of 0.58% for HSV-1 and 2.53 for HSV-2 [10]. In contrast to our findings that demonstrated similar rates of HSV-1 and HSV-2 in women genitalia, they reported that HSV-2 is more frequent in women than HSV-1 (3.81% vs. 0.23%, respectively). The Korean study was based on survey samples tested for sexually transmitted infections (STIs), which makes both their cohort composition and the purpose for testing different from ours. Compatible with our study, they also reported a higher rate of HSV-2 in women compared with men (3.04% vs. 1.4%, respectively).

We have found that HSV-2 infections were much more prevalent among women compared with men, mainly in genital swabs but also at skin samples, which is compatible with the literature which shows that more women are infected with HSV-2 than men worldwide, with the ratio of the prevalence between genders varying across geographical areas [6,11].

Several explanations have been proposed for the higher HSV-2 infection rates in women. The female reproductive tract has a larger surface area compared to the male reproductive tract. Furthermore, there is a higher vulnerability to contagion through the mucosal lining of the women’s external genitalia compared with men’s external genitalia which is mostly covered with keratinized skin [4]. Finally, studies have shown that women are tested more frequently than men [12]. Indeed, in our study, there was a predominance of women among the patients (6390/9189).

Oral sex is considered, by the published literature, to be the mechanism responsible for the presence of HSV-1 in the genitalia [4]. This route of infection could have also allowed for the travel of HSV-2 to the orofacial region. Interestingly, this was not the case, and HSV-2 infections were rarely found in the orofacial region in our study neither in women nor in men. These results are compatible with the published literature. Löwhagen et al. reported that only 4% of 631 orofacial isolates were HSV-2 positive, while 96% were HSV-1 positive [13]. Also, the HERPIMAX study on orofacial herpes simplex virus infections in France concluded that HSV-2 infections do not produce orofacial lesions [14].

Our results and others suggest that HSV-2 is limited in its ability to successfully infect the orofacial epithelia and that both serotypes can readily establish genital infections. The reason for this difference is still an enigma.

Plotting the percentage of positive cases versus age for each gender revealed different curve shapes between genders. Women display a bell-shaped curve with a peak between the ages of 19 and 37 for HSV-1 and a broader peak between the ages of 18 and 48 for HSV-2 (Figure 1C,D). This result is consistent with the finding that the mean age for HSV-2 acquisition in our cohort of hospitalized women was 31.5 for HSV-1 and 41.2 for HSV-2 (*p* < 0.0001, Table 2). In men, both HSV types display sparse acquisition with no age-defined peak (Figure 1A,B). In both genders, HSV-1 was detected in early childhood, while HSV-2 infection was detected, like other sexually transmitted diseases, at the age of 16 years or later, which may be related to the initiation of sexual relations and/or the effect of sex hormones, especially progesterone, on HSV-2 replication in women, as will be discussed later.

The analysis of 287 medical records of hospitalized women with either HSV-1 (161 files) or HSV-2 (126 files) revealed that genital infections with HSV-2 differ from genital infections with HSV-1 in three aspects. We have found that the median age of women infected with HSV-2 is higher compared with HSV-1 and that HSV-2 tends to reactivate more frequently compared with HSV-1. These findings are compatible with the literature [15,16,17].

We also found that pregnancies were more frequent in women with genital HSV-2 infections compared with women with genital HSV-1 infections (Table 2 and Table 3), indicating that pregnancy may serve as a risk factor specifically for HSV-2 reactivation in women that are carriers of latent infection.

We would like to propose that progesterone, which increases during pregnancy, might contribute specifically to HSV-2 reactivation in women during pregnancy. Indeed, the effect of progesterone on the ability of HSV-2 to thrive has been well demonstrated in both animal and in vitro models. Bujko et al. investigated the effect of pregnancy and parenterally administered progesterone on vaginal HSV-2 infection in mice. The susceptibility to vaginal HSV-2 infection among pregnant and progesterone-treated animals was significantly increased compared with non-pregnant, untreated control mice [18]. Recently, the infection of mice early in gestation was demonstrated to be a relevant animal model for investigating outcome of primary HSV-2 infection during pregnancy [19]. A cell culture study using immortalized vaginal epithelial cells (line Vk2) demonstrated increased levels of HSV-2 infection in cells cultured with progesterone compared to cells cultured with estradiol [20]. Many studies have demonstrated that progesterone and estrogen can modify antiviral immune function and alter susceptibility to sexually transmitted viruses [21,22]. Estradiol is generally protective against sexually transmitted viral infections, and progesterone and progestin-based hormonal contraceptives are associated with increased susceptibility [23]. Medroxyprogesterone acetate (MPA), a first-generation synthetic progestin used by over 100 million women worldwide, has been shown to increase the likelihood of HSV-2 infection in rodent and non-human primate studies [24]. Serological studies in humans showed higher HSV-2 seroprevalence in a cohort of women that used progesterone-containing contraception [25].

The enhanced effect of progesterone on HSV-2 infection compared with HSV-1 in animal models might be due to the immunosuppressive capabilities of progesterone which are perhaps needed for HSV-2 to establish infection while HSV-1 might be more robust in its capability to infect, and therefore resides in both the genital and oral epithelium. Alternatively, progesterone may influence gene expression either of the viral or of the host, which sets the stage specifically for HSV-2 infection or propagation. Such an effect was shown for HIV. An in vitro study showed that progesterone decreased CCR5 expression and increased CXCR4 expression in PBMCs of samples of patients both infected and uninfected with, suggesting that progesterone may modulate HIV infection at the receptor level by increasing the expression of HIV co-receptor CXCR4 [26,27]. Investigations exploring gene expression in the presence and absence of progesterone in various viral replicating models, as well as those assessing the relationship between estrogen and progesterone, are needed to understand the role of sex hormones in HSV-2 infections.

Considering the published data, we hypothesize that the high frequency of HSV-2 reactivations associated with pregnancies in our cohort may result from the elevated progesterone levels. Progesterone is extensively used in assisted reproductive technologies and in pregnancy maintenance [28]. In these cases, there is room to consider testing for HSV-2 latent infection prior to treatment. HSV-1 seroprevalence reaches 50–70% in developed countries and 100% in developing countries and HSV-2 seroprevalence varies from 10 to 40% [29].

Studies show that only 10–25% of people with HSV-2 antibodies are aware that they have genital herpes [30,31]. Identifying women with latent HSV-2 infection will allow for better supervision and reactivation prevention and can help prevent infections in neonates and infants.

## 5. Conclusions

The presented findings suggest an association between pregnancy and HSV-2 reactivation (odds ratio = 3.52), which may be related to the elevated level of progesterone during pregnancy.

Pregnant women latently infected with HSV-2 should be aware of possible reactivation episodes during pregnancy. The lack of progesterone in men might also contribute, among other factors, to the difference in HSV-2 prevalence between genders.

## Figures and Tables

**Table 1:** HSV-1 and HSV-2 infections according to gender and site of infection.

Sample Area	Gender	Number of Tests	HSV-1 Positive N (%)	* *p* Value Significance Level 0.05	HSV-2 Positive N (%)	* *p* Value Significance Level 0.05
Orofacial	Men	1229	342 (27.8)	*p* = 0.96998	12 (0.9)	*p* = 0.732791
Orofacial	Women	1070	297 (27.7)		12 (1.1)	
Other sites (skin)	Men	1402	303 (21.6)	*p* = 0.00738	61 (4.3)	*p* < 0.00001
Other sites (skin)	Women	3241	591 (18.2)		327 (10.0)	
Genital	Men	168	25 (14.8)	*p* = 0.710524	12 (7.1)	*p* = 0.008604
Genital	Women	2079	332 (15.9)		300 (14.4)	

* *p* value was calculated by the Chi-Square test for association between two categorical variables.

**Table 2:** HSV-1 and HSV-2 genital infection among women hospitalized in SMC (N = 287).

Characteristic	HSV-1 (*n* = 161)		HSV-2 (*n* = 126)		*p* Value
Age mean ± SD	31.5	±14.1	41.2	±17.3	*p* < 0.0001
Primary infection N (%)	25	(15.6)	4	(3.2)	*p* = 0.0005
Pregnancy N (%)	20	(12.4)	35	(27.8)	*p* = 0.001
Miscarriage six months prior to infection N (%)	0	0	4	(3.2)	*p* = 0.0232
Birth control use N (%)	59	(36.6)	18	(14.3)	*p* < 0.0001
IVF in the past N (%)	9	(5.6)	6	(4.8)	*p* = 0.7662
Background of malignancy N (%)	18	(11.2)	18	(14.4)	*p* = 0.4155

**Table 3:** Multivariate logistic regression model for genital HSV-2 infection among women (N = 287).

Variable	Odds Ratio	95% CI	*p* Value
Age	1.23	1.13–13.4	*p* < 0.0001
Non-primary infection	4.1	1.34–12.57	*p* = 0.0136
Pregnancy	3.52	1.84–6.73	*p* = 0.0001

## Data Availability

All data are available within the manuscript.

## References

[1] Salameh S., Sheth U., Shukla D. (2012). Early events in herpes simplex virus lifecycle with implications for an infection of lifetime. Open Virol. J..

[2] Ryder N., Jin F., McNulty A.M., Grulich A.E., Donovan B. (2009). Increasing role of herpes simplex virus type 1 in first-episode anogenital herpes in heterosexual women and younger men who have sex with men, 1992–2006. Sex. Transm. Infect..

[3] Wald A. (2006). Genital HSV-1 infections. Sex. Transm. Infect..

[4] Looker K.J., Magaret A.S., Turner K.M., Vickerman P., Gottlieb S.L., Newman L.M. (2015). Global estimates of prevalent and incident herpes simplex virus type 2 infections in 2012. PLoS ONE.

[5] Mihimit A., Adawaye C., Péré H., Costiniuk C., Koyalta D., Mbopi-Keou F.-X., Bouassa R.-S.M., Talla F., Moussa S., Longo J.D.D. (2020). HSV-2 Infection as a Potential Cofactor for HIV Disease Progression and Selection of Drug Resistance Mutations in Adults under WHO-Recommended First-Line Antiretroviral Therapy: A Multicentric, Cross-Sectional Study in Cameroon, Central African Republic, Chad, and Gabon. Trop. Med. Infect. Dis..

[6] Grayo S. (2021). Is the ZIKV Congenital Syndrome and Microcephaly Due to Syndemism with Latent Virus Coinfection?. Viruses.

[7] Anzivino E., Fioriti D., Mischitelli M., Bellizzi A., Barucca V., Chiarini F., Pietropaolo V. (2009). Herpes simplex virus infection in pregnancy and in neonate: Status of art of epidemiology, diagnosis, therapy and prevention. Virol. J..

[8] Filén F., Strand A., Allard A., Blomberg J., Herrmann B. (2004). Duplex real-time polymerase chain reaction assay for detection and quantification of herpes simplex virus type 1 and herpes simplex virus type 2 in genital and cutaneous lesions. Sex. Transm. Dis..

[9] McQuillan G., Kruszon-Moran D., Flagg E.W., Paulose-Ram R. (2018). Prevalence of Herpes Simplex Virus Type 1 and Type 2 in Persons Aged 14–49: United States, 2015–2016. NCHS Data Brief.

[10] Oh E.J., Yuk Y.S., Kim J.K. (2021). Laboratory investigations of herpes simplex virus-1 and -2 clinical samples in Korea. Osong Public Health Res. Perspect..

[11] Looker K.J., Garnett G.P., Schmid G.P. (2008). An estimate of the global prevalence and incidence of herpes simplex virus type 2 infection. Bull. World Health Organ..

[12] Friberg I.O., Krantz G., Määttä S., Järbrink K. (2016). Sex differences in health care consumption in Sweden: A register-based cross-sectional study. Scand. J. Public Health.

[13] Löwhagen G.-B., Tunbäck P., Bergström T. (2002). Proportion of herpes simplex virus (hsv) type 1 and type 2 among genital and extragenital hsv isolates. Acta Derm.-Venereol..

[14] Malvy D., Ezzedine K., Lancon F., Halioua B., Rezvani A., Bertrais S., El Hasnaoui A. (2007). Epidemiology of orofacial herpes simplex virus infections in the general population in France: Results of the HERPIMAX study. J. Eur. Acad. Dermatol. Venereol..

[15] Daniels B., Wand H., Ramjee G. (2016). Mdp the MDP Team Prevalence of Herpes Simplex Virus 2 (HSV-2) infection and associated risk factors in a cohort of HIV negative women in Durban, South Africa. BMC Res. Notes.

[16] Wald A., Zeh J., Selke S., Ashley R.L., Corey L. (1995). Virologic characteristics of subclinical and symptomatic genital herpes infections. N. Engl. J. Med..

[17] Garland S.M., Steben M. (2014). Genital herpes. Best Pract. Res. Clin. Obstet. Gynaecol..

[18] Bujko M., Sulović V., Zivanović V., Lako B., Dotlić R. (1988). Effect of progesterone and pregnancy on the replication of herpes simplex virus type 2 in vivo. Clin. Exp. Obstet. Gynecol..

[19] Felker A.M., Nguyen P., Kaushic C. (2021). Primary HSV-2 Infection in Early Pregnancy Results in Transplacental Viral Transmission and Dose-Dependent Adverse Pregnancy Outcomes in a Novel Mouse Model. Viruses.

[20] Lee Y., Dizzell S.E., Leung V., Nazli A., Zahoor M.A., Fichorova R.N., Kaushic C. (2016). Effects of Female Sex Hormones on Susceptibility to HSV-2 in Vaginal Cells Grown in Air-Liquid Interface. Viruses.

[21] Kaushic C., Ashkar A.A., Reid L.A., Rosenthal K.L. (2003). Progesterone increases susceptibility and decreases immune responses to genital herpes infection. J. Virol..

[22] Wira C.R., Rodriguez-Garcia M., Patel M.V. (2015). The role of sex hormones in immune protection of the female reproductive tract. Nat. Rev. Immunol..

[23] Kaushic C., Roth K.L., Anipindi V., Xiu F. (2011). Increased prevalence of sexually transmitted viral infections in women: The role of female sex hormones in regulating susceptibility and immune responses. J. Reprod. Immunol..

[24] Gillgrass A.E., Ashkar A.A., Rosenthal K.L., Kaushic C. (2003). Prolonged exposure to progesterone prevents induction of protective mucosal responses following intravaginal immunization with attenuated herpes simplex virus type 2. J. Virol..

[25] Grabowski M.K., Gray R.H., Makumbi F., Kagaayi J., Redd A.D., Kigozi G., Tobian A.A. (2015). Use of injectable hormonal contraception and women’s risk of herpes simplex virus type 2 acquisition: A prospective study of couples in Rakai, Uganda. Lancet Glob. Health.

[26] Cabrera-Muñoz E., Fuentes-Romero L.L., Zamora-Chávez J., Camacho-Arroyo I., Soto-Ramírez L.E. (2012). Effects of progesterone on the content of CCR5 and CXCR4 coreceptors in PBMCs of seropositive and exposed but uninfected Mexican women to HIV-1. J. Steroid Biochem. Mol. Biol..

[27] Murakami T., Yamamoto N. (2010). Role of CXCR4 in HIV infection and its potential as a therapeutic target. Future Microbiol..

[28] Bulleti C., Bulleti F.M., Sciorio R., Guido M. (2022). Progesterone: The Key Factor of the Beginning of Life. Int. J. Mol. Sci..

[29] LeGoff J., Péré H., Bélec L. (2014). Diagnosis of genital herpes simplex virus infection in the clinical laboratory. Virol. J..

[30] Fleming D.T., McQuillan G.M., Johnson R.E., Nahmias A.J., Aral S.O., Lee F.K., St. Louis M.E. (1997). Herpes simplex virus type 2 in the United States, 1976–1994. N. Engl. J. Med..

[31] Leone P., Fleming D.T., Gilsenan A.W., Li L., Justus S. (2004). Seroprevalence of herpes simplex virus-2 in suburban primary care offices in the United States. Sex. Transm. Dis..

